# Utility of interferon-γ ELISPOT assay responses in highly tuberculosis-exposed patients with advanced HIV infection in South Africa

**DOI:** 10.1186/1471-2334-7-99

**Published:** 2007-08-28

**Authors:** Stephen D Lawn, Nonzwakazi Bangani, Monica Vogt, Linda-Gail Bekker, Motasim Badri, Marjorie Ntobongwana, Hazel M Dockrell, Robert J Wilkinson, Robin Wood

**Affiliations:** 1 The Desmond Tutu HIV Centre, Institute of Infectious Disease and Molecular Medicine, Faculty of Health Sciences, University of Cape Town, South Africa; 2 Clinical Research Unit, Department of Infectious and Tropical Diseases, London School of Hygiene & Tropical Medicine, London, UK; 3 Immunology Unit, Department of Infectious and Tropical Diseases, London School of Hygiene & Tropical Medicine, London, UK; 4 Institute of Infectious Disease & Molecular Medicine and Department of Medicine, University of Cape Town, South Africa; 5 Wellcome Trust Centre for Research in Clinical Tropical Medicine, Division of Medicine, Imperial College London, UK

## Abstract

**Background:**

Interferon-gamma (IFN-γ) ELISPOT assays incorporating *Mycobacterium tuberculosis*-specific antigens are useful in the diagnosis of tuberculosis (TB) or latent infection. However, their utility in patients with advanced HIV is unknown. We studied determinants of ELISPOT responses among patients with advanced HIV infection (but without active TB) living in a South African community with very high TB notification rates.

**Methods:**

IFN-γ responses to ESAT-6 and CFP-10 in overnight ELISPOT assays and in 7-day whole blood assays (WBA) were compared in HIV-infected patients (HIV+, n = 40) and healthy HIV-negative controls (HIV-, n = 30) without active TB. Tuberculin skin tests (TSTs) were also done.

**Results:**

ELISPOTs, WBAs and TSTs were each positive in >70% of HIV- controls, reflecting very high community exposure to *M. tuberculosis*. Among HIV+ patients, quantitative WBA responses and TSTs (but not the proportion of positive ELISPOT responses) were significantly impaired in those with CD4 cell counts <100 cells/μl compared to those with higher counts. In contrast, ELISPOT responses (but not WBA or TST) were strongly related to history of TB treatment; a much lower proportion of HIV+ patients who had recently completed treatment for TB (n = 19) had positive responses compared to those who had not been treated (11% versus 62%, respectively; P < 0.001). Multivariate analysis confirmed that ELISPOT responses had a strong inverse association with a history of recent TB treatment (adjusted OR = 0.06, 95%CI = 0.10–0.40, P < 0.01) and that they were independent of CD4 cell count and viral load. Among HIV+ individuals who had not received TB treatment both the magnitude and proportion of positive ELISPOT responses (but not TST or WBA) were similar to those of HIV-negative controls.

**Conclusion:**

The proportion of positive ELISPOT responses in patients with advanced HIV infection was independent of CD4 cell count but had a strong inverse association with history of TB treatment. This concurs with the previously documented low TB risk among patients in this cohort with a history of recent treatment for TB. These data suggest ELISPOT assays may be useful for patient assessment and as an immuno-epidemiological research tool among patients with advanced HIV and warrant larger scale prospective evaluation.

## Background

The HIV-associated tuberculosis (TB) epidemic in sub-Saharan Africa is fuelling a global increase in TB incidence of 1% per year [1]. TB incidence rates in southern Africa have reached almost unprecedented levels [2] and much of this disease remains undetected in the community [3]. This escalating epidemic led to the declaration by the World Health Organisation in 2005 of "a regional emergency requiring urgent and extraordinary actions" [4]. However, most existing tools with which to confront the TB epidemic are blunt, especially those used for diagnosis of *Mycobacterium tuberculosis *infection and disease in HIV-infected patients.

Recent developments of immune-based assays to detect *Mycobacterium tuberculosis *infection are a significant advance [5]. ESAT-6 and CFP-10 are two proteins encoded by the RD1 genomic segment of *M. tuberculosis*, which is absent from all BCG strains and the vast majority of environmental mycobacteria [6-8]. As a result, enzyme-linked immunospot (ELISPOT) assays that detect interferon-gamma (IFN-γ) release in response to these antigens differentiate between *M. tuberculosis *infection and immune sensitisation by BCG vaccination or exposure to environmental mycobacteria. In outbreaks of TB in the UK, ELISPOT responses among contacts showed better correlation with the degree of exposure than tuberculin skin tests (TSTs) [9,10]. Among HIV-negative patients with culture-positive TB, ELISPOT assays have a sensitivity of approximately 80–90% [11-13]. Moreover, increasing evidence suggests that ELISPOT responses in human and bovine models correlate with mycobacterial load during antituberculosis treatment [13-18].

At present, very few studies have examined the utility of ELISPOT assays in HIV-infected individuals. In a study from Zambia, ELISPOT responses to ESAT-6 or CFP-10 were positive in 90% (n = 39) of HIV-infected patients with sputum smear-positive pulmonary TB [19]. Also, when used in the diagnosis of TB in South African children, the sensitivity of the assay was not significantly impaired by HIV coinfection [12]. More recently the assay was found to be relatively unimpaired in the detection of either latent *M. tuberculosis *infection or active TB in patients with moderately advanced HIV infection [20,21]. However, responses in those with advanced HIV have not previously been reported.

The aim of the present study was to identify determinants of ELISPOT responses among patients with advanced HIV infection (but not active TB) living in a South African community with very high TB incidence. Overnight IFN-γ ELISPOT assay responses were assessed among a group of HIV-infected patients enrolling in an antiretroviral treatment service and were compared with responses in a group of healthy controls living in the same community. To provide greater insight, these responses were compared to 7-day whole blood assays (WBA) of IFN-γ release and TSTs.

## Methods

HIV-infected (HIV+) patients were recruited to the study at the antiretroviral treatment clinic based at the Gugulethu Community Health Centre in Cape Town. The study cohort and clinic have previously been described in detail [22-25]. The district has a predominantly African population of over 300,000, the vast majority of whom live in conditions of low socioeconomic status. In 2003 the antenatal HIV seroprevalence was 28% and the annual TB notification rate exceeded 1,000/100,000 [26]. Patients were referred to the ART programme for evaluation for eligibility for antiretroviral treatment under national guidelines.

Consecutive HIV+ patients were recruited to the study before starting antiretroviral treatment. Non-pregnant adults between the ages of 18 and 50 years, who were not currently receiving antituberculosis treatment nor had evidence of active TB were eligible. Details of previous medical history were obtained from the referral letter and were cross-checked with the patient. Patients were clinically characterised and carefully evaluated for TB. Available investigations for TB included sputum smear microscopy and liquid culture (MGIT, Becton Dickinson, Sparks, Maryland, USA), chest radiology, and fine needle aspiration of lymphadenopathy for cytology and culture. Nebulised sputum induction was accessible when required. Patients with active TB diagnosed at baseline were excluded as were those who subsequently developed TB within 4 months of the recruitment date since they may have had active sub-clinical TB at the time of testing. Standard antituberculosis treatment nationally uses a 6-month rifampicin-containing regimen and primary and secondary isoniazid prophylaxis is not recommended by the national TB control programme.

Healthy HIV- control patients aged 18–50 years were recruited from the same community. These individuals were part of a prospective cohort being studied in preparation for phase III HIV vaccine trials. Consecutive individuals with two negative HIV tests three months apart who remained asymptomatic and had no previous history of TB or other significant morbidity were enrolled.

At a single clinic visit venous blood was taken and TSTs were done using 2 TU purified protein derivative (PPD) RT23 (Statens Serum Institut, Copenhagen, Denmark) on the volar aspect of the forearm. TSTs were read by a single health care worker experienced in the technique, measuring the diameter of induration at 48–72 hours using callipers. Plasma HIV-1 load was measured using Versant™ HIV-1 RNA 3.0 branched chain DNA assay (Bayer HealthCare, Leverkusen, Germany) and blood CD4 cell counts were measured by flow cytometry using FACSCount™ (Becton Dickinson Inc., Franklin Lakes, NJ, USA). All subjects studied gave written informed consent. The study was approved by the Research Ethics Committee of the University of Cape Town and conformed to the declaration of Helsinki.

### ELISPOT assays

Peripheral blood mononuclear cells (PBMCs) were separated from heparinized venous blood by Ficoll-Paque centrifugation. A commercially available IFN-γ ELISPOT kit containing a pre-coated 96-well plate was used (Mabtech, Stockhom, Sweden). Paired wells with 250,000 cells/well were stimulated with anti-CD3 antibody at 100 ng/ml (positive control, Mabtech), were left unstimulated (negative control) or contained the following antigens each at 5 μg/ml final concentration: PPD RT49 (Statens Serum Institut) or recombinant ESAT-6 and CFP-10 (Lionex, Braunschweig, Germany). After incubation for 18 hours, plates were developed according to the manufacturer's protocol. Plates were read on an Immunospot Series 3B Analyzer (Cellular Technology, Cleveland, OH, USA) and were retained for visual inspection in the case of anomaly. Low level spot counts in unstimulated negative control wells were subtracted from the test well results. The mean number of spot-forming units (SFU) in paired wells was multiplied by 4 to provide SFU/10^6 ^PBMC. Counts of ≥ 20 SFU/10^6 ^PBMC that were at least ≥ 2-fold greater than background counts were predefined as positive responses. Using this cut-off, this assay produces very similar results to other commercially available and 'in-house' assays performed in the same laboratory [20,21].

### Whole blood assays

Whole blood assays were done using a methodology similar to that described previously [27,28]. Heparinized whole blood was diluted 5-fold using RPMI 1640 supplemented with penicillin, streptomycin and 2 mM L-glutamine and was plated into 96-well plates in the presence of antigen or mitogen or left unstimulated. Phytohaemagglutinin (PHA, Sigma, St. Louis, MS, USA), PPD RT49, and recombinant ESAT-6 and CFP-10 were all used at a final concentration of 5 μg/ml. Any small residual traces of endotoxin in antigen preparations were neutralised using 10 μg/ml polymyxin B sulphate (Sigma) as verified in pilot experiments. Day 7 supernatants were harvested following incubation at 37°C. All assays were piloted and conditions, antigen concentrations and time-points were optimised using patient and control samples. IFN-γ responses to recombinant ESAT-6 were equivalent when compared with those generated using a sample of recombinant antigen from Statens Serum Institut, Copenhagen.

Concentrations of IFN-γ were determined in paired samples of supernatant using an ELISA with a standard curve that ranged from 10,000 pg/ml to 41 pg/ml and using previously described methodology [28,29]. Background concentrations of IFN-γ were subtracted from sample results, which were then categorised as positive if ≥2-fold higher that the lower limit of detection of the assay. Negative control values falling below the lower limit of detection were assigned half the value of the lowest standard.

All laboratory assays were done blinded to the status of the patients.

### Data analysis

χ^2^, Fisher's exact and Mann-Whitney U tests were used to compare proportions and medians as appropriate. Assay responses were evaluated as continuous (quantitative) variables and as categorical variables (positive or negative responses) according to the thresholds stated above. Associations between patient characteristics and positive ELISPOT and WBAs were examined using univariate analyses and multiple logistic regression models. CD4 cell count, plasma viral load and history of previous TB were included in the multivariate models *a priori*; age and sex were included if there was a trend towards an association (p < 0.10). SAS version 8.2 (SAS, Cary, North Carolina, USA) and Prism version 4.0 (GraphPad Software, San Diego, CA, USA) software were used for data analysis.

## Results

### Patient characteristics

A total of 77 subjects were recruited; 7 were excluded due to failed ELISPOT assays (n = 4; no positive control response or technical error) or the development of possible TB (n = 3). Eleven individuals invited to participate declined (predominantly due to transportation difficulties). Among those included in the analysis, 40 were HIV+ patients being evaluated for antiretroviral treatment; these had baseline characteristics (Table 1) which were similar to those described in previous reports of this treatment cohort [22-24]. None of the patients had serious co-morbidity (such as lymphoma, Kaposi's sarcoma requiring cytotoxic chemotherapy or diabetes mellitus) that might have affected T cell assays. CD4 cell counts showed that immunodeficiency was advanced in most patients; 48% had a history of completed treatment for TB within the preceding 3 years (Table 1) consistent with previous findings [22]. The median period since completion of TB treatment was 4 months (IQR, 2–13 months). HIV- controls (n = 30) had similar age and sex distribution as HIV+ patients (Table 1).

**Table 1:** Characteristics of HIV-negative controls (HIV-) and HIV-infected patients (HIV+)

**Characteristic**	**HIV- controls**	**HIV+ patients**		
		
		**All**	**With history of TB**	**With no history of TB**
Number	30	40	19	21
Median age (years)	28	31	30	32
Female	23 (77)	32 (80)	13 (68)	19 (90)
CD4 count (cells/μL)				
Median (IQR)	-	114 (72–246)	98 (44–160)	117 (77–267)
Distribution				
0–99	-	17 (43)	10 (53)	7 (33)
100–199	-	10 (25)	5 (26)	5 (24)
≥200	-	13 (32)	4 (21)	9 (43)
Median viral load		5.0 (4.5–5.3)	5.0 (4.6–5.4)	4.9 (4.4–5.3)
TST (mm)				
0	6 (20)	23 (58)	13 (68)	10 (48)
1–9	1 (3)	0 (0)	0 (0)	0 (0)
≥100	23 (77)	17 (43)	6 (32)	11 (52)

Unless otherwise stated, values show numbers (%) of patients.

Viral load in log copies/mL. TST = tuberculin skin test

A large proportion (58%) of the HIV+ patients had cutaneous anergy to PPD (Table 1) and TSTs reactions >10 mm occurred in a significantly lower proportion of HIV+ patients than HIV- controls (43% versus 77%, P < 0.05). TST responses were significantly associated with CD4 cell count; the proportion of patients with a TST response >10 mm was much lower among those with CD4 cell counts <100 cells/μl (n = 17) compared to those with CD4 cell counts >100 cells/μl (n = 23) (16% versus 56%, respectively; P < 0.05). In contrast, TST responses were not significantly associated with history of TB treatment (P = 0.2).

### ELISPOT and WBA results

In all 70 subjects included in the analysis the positive control stimuli produced measurable responses in ELISPOT assays and WBAs. The magnitude of responses to all stimuli were significantly lower among HIV+ patients compared to HIV- controls (Table 2). However, in contrast to TSTs, the magnitude of these responses in HIV+ patients was not significantly associated with CD4 cell count using either assay. Instead IFN-γ responses, especially ELISPOT results, were strongly associated with history of previous TB treatment; responses among treated patients were lower than those who had no history of treatment (Table 2).

**Table 2:** Quantitative interferon-γ (IFN-γ) responses to anti-CD3 (positive control) and mycobacterial antigens as assessed by ELISPOT assay and 7-day whole blood assay (WBA)

**Patient characteristics**	**ELISPOT^a^**				**WBA^b^**			
		
	**Anti-CD3**	**PPD**	**ESAT-6**	**CFP-10**	**PHA**	**PPD**	**ESAT-6**	**CFP-10**
**HIV- (n = 30)**	1723 (846–2471)	61 (26–214)	24 (7–199)	30 (6–89)	>10,000	>10,000	4820	3678
**HIV+ (n = 40)**	626 (398–2015)	21 (2–53)	7 (2–46)	6 (0–39)	3991	799	355	96
**P value**	0.012	0.001	0.004	0.010	<0.001	<0.001	<0.001	<0.001

**HIV+ CD4>100^c ^(n = 23)**	654 (290–2296)	30 (4–64)	16 (2–58)	30 (0–82)	4213	616	34	49
**HIV+ CD4<100 (n = 17)**	600 (400–1912)	8 (2–49)	2 (1–17)	1 (1–10)	2550	1034	528	125
**P value**	0.93	0.42	0.19	0.12	0.13	0.89	0.14	0.12

**HIV+ no previous TB Rx^d ^(n = 19)**	476 (269–2299)	34 (3–147)	22 (2–80)	36 (1–80)	6938	1529	495	179
**HIV+ previous TB Rx (n = 21)**	1020 (400–1844)	6 (2–42)	5 (0–14)	0 (0–6)	2749	313	65	20
**P value**	0.70	0.085	0.044	0.028	0.10	0.115	0.220	0.038

^a^ELISPOT responses expressed as median (interquartile range) spot-forming units/10^6 ^peripheral blood mononuclear cells.

^b^WBA responses expressed as median values in pg/mL.

^c^CD4 cell count in cells/μL.

^d^TB Rx = treatment for tuberculosis

**Figure 1:**
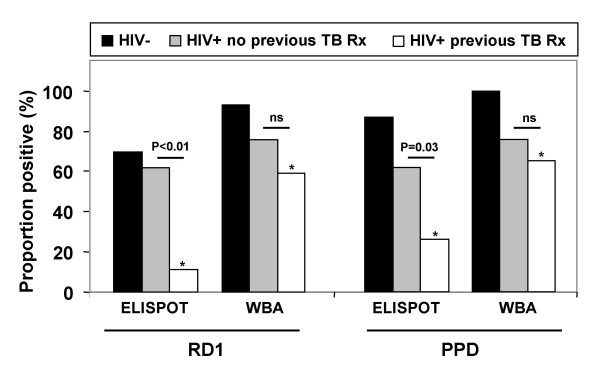
Proportion of positive IFN-γ ELISPOT responses and 7-day IFN-γ whole blood assay (WBA) responses to ESAT-6 and/or CFP-10 (RD1) and purified protein derivative (PPD) among HIV- controls (n = 30) and HIV+ patients (n = 40) subdivided into those with (n = 19) or without (n = 21) a history of previous tuberculosis treatment (TB Rx). ELISPOT assay responses in HIV+ patients were strongly associated with history of TB treatment. *In HIV+ patients who had previously received TB treatment, the proportions of positive responses to RD1 and PPD antigens were significantly lower using the ELISPOT assay than the WBA (P < 0.05 for each comparison). ns = not significant.

We next calculated the proportions of patients with assay responses categorised as positive according to the predefined thresholds (Figure 1). In general, greater proportions of 7-day WBA responses were positive compared to the overnight ELISPOT assay. Furthermore, the proportions of positive responses were typically greater using PPD compared to RD1 antigens.

Positive responses to RD1 antigens were detected in 70% of healthy HIV- controls using the ELISPOT assay and in a significantly greater proportion using the 7-day WBAs (93%; P < 0.05) (Figure 1). Positive responses to PPD were also observed in very high proportions of HIV- controls using both assays (Figure 1). Similar to the quantitative responses (Table 2), the proportions of positive ELISPOT responses in HIV+ patients were also very strongly associated with previous history of TB treatment (Figure 1). Compared to those who had not previously received TB treatment, treated patients had a 5.6-fold lower proportion of positive ELISPOT responses to RD1 antigens (P < 0.01) and a 2.4-fold lower proportion of positive ELISPOT responses to PPD (P = 0.02) (Figure 1). In marked contrast, WBA responses in HIV+ patients did not show a significant inverse association with history of TB treatment (P > 0.25 for each comparison) (Figure 1). Thus, there was a clear dissociation between ELISPOT responses and WBA responses in those who had previously been treated for TB (Figure 1).

**Figure 2:**
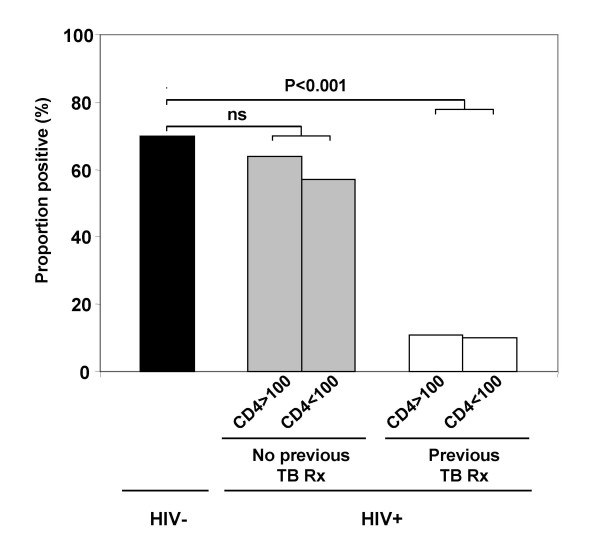
Proportion of positive ELISPOT IFN-γ responses to *M. tuberculosis*-specific antigens (ESAT-6 and/or CFP-10) among HIV- controls (n = 30) compared to HIV+ patients (n = 40) stratified by CD4 cell count >100 cells/μl (n = 23) or <100 cells/μl (n = 17) and according to history of recent TB treatment (n = 19) or no history of such treatment (n = 21). ns = not significant

We next examined the effect of CD4 cell count on the proportion of positive assay responses by comparing the results of those with CD4 cell counts <100 cells/μl (n = 17) with those of patients with CD4 cell counts >100 cells/μl (n = 23). There was no significant difference between these groups using the WBA (82% vs 61%, respectively; P = 0.18) although there was a non-significant trend towards an association using the ELISPOT assay (24% vs 52%, respectively; P = 0.10). Importantly, however, history of TB treatment is confounded as a variable by CD4 cell count in this cohort [22] (Table 1). Thus, we next analysed ELISPOT responses stratified by both CD4 cell count and history of TB treatment. This clearly showed that responses were strongly associated with history of TB treatment but not with CD4 cell count (Figure 2).

To confirm these findings we next did multivariate analysis to identify factors independently associated with positive ELISPOT responses to RD1 antigens (Table 3). This showed that ELISPOT responses were not significantly associated with CD4 cell count or viral load but that there was a highly significant independent association with history of TB treatment.

**Table 3:** Multivariate analysis of factors associated with positive IFN-γ ELISPOT responses to ESAT-6 or CFP-10 in HIV+ patients (n = 40)

**Variable**		**Crude OR**	**P value**	**Adjusted OR**	**P value**
**Age**	<31 years	1.00		-	-
	>31 years	0.58 (0.16–2.12)	0.41	-	-
**Sex**	Male	1.00		-	-
	Female	5.44 (0.60–49.56)	0.13	-	-
**CD4 count**	>100	1.00		1.00	
**(cells/μL)**	<100	0.34 (0.08–1.34)	0.12	0.58 (0.11–3.16)	0.53
**Log viral load**	<5.0	1.00			
**(copies/mL)**	>5.0	0.39 (0.10–1.49)	0.17	0.30 (0.05–1.69)	0.17
**History of TB**	No	1.00			
	Yes	0.07 (0.01–0.40)	<0.01	0.06 (0.10–0.40)	<0.01

OR = odds ratio

### Responses in HIV+ patients with no history of TB treatment

Results thus far indicated that HIV+ patients who did not have a history of recent TB treatment were much more likely to have positive ELISPOT responses to RD1 antigens than those who had been treated. We reasoned that while treated patients were likely to have cleared any viable mycobacteria, untreated patients would have a similar risk of latent *M. tuberculosis *as HIV- controls. We therefore compared IFN-γ responses in HIV- controls and the sub-group of HIV+ patients with no history of TB treatment. There was indeed no significant difference between these groups in quantitative ELISPOT responses to PPD, ESAT-6 and CFP-10 (P > 0.1 for all comparisons) whereas quantitative WBA responses were much lower in the HIV+ subgroup (P < 0.01 for all stimuli) (Table 2). Moreover, the proportion of positive ELISPOT responses did not significantly differ between the HIV- and the untreated HIV+ subgroup (P = 0.55) (Figure 2).

## Discussion

This study conducted in a setting with very high TB burden is, to our knowledge, the first to assess the utility of ELISPOT responses to *M. tuberculosis*-specific antigens among HIV-infected patients with advanced immunodeficiency. To provide further insight into the determinants of such responses, we compared the overnight ELISPOT assay (which assesses rapid effector memory T cell responses [18,30,31] with a 7-day IFN-γ WBA (which reflects central memory T cell responses [32]. We found that both assays were able to detect IFN-γ responses to ESAT-6 and CFP-10 in a substantial proportion of HIV+ patients despite the low median CD4 cell count of 114 cells/μl. The proportion of positive ELISPOT responses was independent of CD4 cell count in stratified and multivariate analyses (Table 2, Figure 2); instead responses were strongly associated with history of TB treatment (Figures 1 and 2; Tables 2 and 3). Almost half the HIV+ patients had completed treatment for TB a median of 4 months earlier, resulting in likely clearance of the burden of viable mycobacterial infection; this was reflected by a low proportion of positive ELISPOT responses in these patients but persistence of a high proportion of positive WBA (memory) responses. In contrast, ELISPOT responses in HIV+ patients with no history of TB treatment did not significantly differ from those of HIV- controls. Collectively, these observations suggest the ELISPOT assay provides data that are clinically and immuno-epidemiologically informative in patients with advanced HIV.

Antigen stimulation in 7-day WBA permits T cells to differentiate and proliferate, leading to amplification of IFN-γ responses. Similar to lymphocyte proliferation assays, WBAs thereby provide an assessment of memory responses to antigens encountered at any time past or present. In contrast, the overnight ELISPOT assay detects rapid effector memory cells; such cells are thought to have recently encountered antigen from viable organisms *in vivo *and can rapidly release IFN-γ when re-exposed to antigen *in vitro *[18,30,31]. Thus, ELISPOT responses appear to be more strongly associated with current antigen load rather than with past antigen exposure [13-18]. These facts provide a logical rationale for the very strong inverse association between ELISPOT responses (but not 7-day WBA responses) and history of recent TB treatment in HIV+ patients.

The HIV- control group was recruited in the same community and had similar demographic characteristics as the HIV+ group and so were likely to have similar prior exposure to *M. tuberculosis*. Retention within an HIV-negative cohort for ≥3 months provided the opportunity to prospectively verify that these patients were free of both TB and HIV. Positive WBA responses to mycobacterial antigens were detected among a very high proportion of HIV- controls, which is consistent with extremely high rates of previous exposure to *M. tuberculosis *in this community. Similar to a study in a neighbouring community in Cape Town [20], TSTs ≥ 10 mm and ELISPOT responses to RD1 antigens were positive in 70% and 77% of HIV- controls, respectively, consistent with high rates of latent *M. tuberculosis *infection.

The HIV+ group was drawn from a very well-characterised cohort of patients enrolling for antiretroviral treatment in which risk factors for TB have been established [22]. An important finding previously reported was that patients who had completed successful TB treatment within the preceding 2 years had an approximately 5-fold lower risk of active TB compared to those who had not [22]. The ELISPOT results in this study strongly concur with this observation. We suggest that successful TB treatment would have cleared viable mycobacterial infection, resulting in low risk of reactivation TB and negative ELISPOT responses due to absence of antigen. An alternative hypothesis to explain the ELISPOT data would be that those who had previously had TB developed lasting suppression of IFN-γ responses to RD1 antigens, although the WBA data do not support this.

Our findings are consistent with previous studies showing that HIV infection does not appear to substantially undermine ELISPOT responses in patients with either active TB [12,19,21] or latent *M. tuberculosis *infection [19-21]. In two separate studies, Rangaka *et al*. found that assay responses to RD1 antigens were not significantly impaired in two cohorts of patients with latent infection and moderate HIV-associated immunodeficiency (both median CD4 cell counts of 392 cells/μl and 464 cells/μl) [20,21]. Our data extend this observation to patients with advanced immunodeficiency (median CD4 cell count = 114 cells/μl). Although we found no impact of CD4 cell count on the proportion of positive responses, there was a trend towards overall quantitative ELISPOT responses being reduced in those with CD4 cell counts <100 cells/μl and the study lacks the statistical power to adequately assess this.

The normalised input of peripheral mononuclear cells used in ELISPOT assays may, in part, explain why this assay appears to retain utility among patients with low CD4 cell counts. This also might explain why studies using a commercially available data overnight IFN-γ whole blood assay (QuantiFERON-TB Gold, Cellestis, Melbourne, Australia) in which the cell input is not normalised have found that responses do not appear to be greatly affected in HIV-infected patients with moderate immunodeficiency but that there was a high rate of indeterminate results among those with advanced immunodeficiency [20,33,34]. It cannot be assumed that only CD4 T cell responses are measured by these assays as other IFN-γ-secreting PBMC subsets such as CD8 T cells and NK cells may also be detected. Future immunophenotypic studies would be useful in this regard.

A high proportion of HIV+ patients had anergic TSTs, confirming the known limited utility of this test in advanced HIV. Moreover, both TST responses and quantitative WBA responses to mycobacterial antigens were very strongly associated with CD4 cell count, contrasting with the ELISPOT assay. A potential reason for this is that both TST and WBA responses depend on interleukin-2-mediated T cell proliferation, which is markedly inhibited by HIV infection [35,36]. In contrast, the overnight ELISPOT assay detects terminally differentiated effector cells [18,30] and is therefore independent of T cell proliferation.

This study has certain limitations. Case control studies may be subject to selection bias. However, the characteristics of the HIV+ group were representative of those previously described in this cohort [22] and HIV- controls had similar demographic characteristics as HIV+ patients and were enrolled within the community rather than at health facilities. The assays used are research tools and are not directly comparable with commercially available IFN-γ-release assays currently used in clinical practice. Although the group of HIV+ patients with a history of TB treatment was heterogeneous with regard to the time since completion of TB treatment, we have previously found that such patients have a substantially lower risk of TB [22]. Thus, history of previous TB treatment was included as a key variable within the analyses. This study, however, was not designed to specifically evaluate the impact of TB treatment on ELISPOT responses and the data should not be over-interpreted in this regard. Common to all such studies, the sensitivity of the ELISPOT assay in diagnosis of *M. tuberculosis *infection in this population cannot be definitively evaluated in the absence of a gold standard. The study included few HIV-infected patients with high CD4 cell counts and this may have limited the ability to fully evaluate the effect of CD4 cell count on ELISPOT responses. However, our findings are consistent with other published data [20,21]. The number of patients studied was limited, but in view of the very high rates of TB exposure in this community and very strong statistical associations observed, the aims of the study were nevertheless achieved.

## Conclusion

This study provides evidence that the IFN-γ ELISPOT assay retains utility among patients with advanced HIV infection as suggested by the following key observations: (i) the proportion of positive responses was not associated with CD4 cell count in stratified and multivariate analyses; (ii) responses were strongly related to history of TB treatment – a known key factor associated with TB risk in this patient population; (iii) responses in HIV+ patients who had not been treated for TB in the past were similar to those of HIV- controls. Collectively these data indicate that the ELISPOT assay provides data that are informative in patients with advanced HIV and is therefore likely to be useful for patient assessment and as an immuno-epidemiological tool. These data provide an important basis for the justification of future large scale evaluation of the assay in prospective cohort studies of patients with advanced HIV.

## Competing interests

The author(s) declare that they have no competing interests.

## Authors' contributions

SDL designed the study, analysed the data and wrote the manuscript. NB and MV did the laboratory analyses. MN obtained clinical data and did the TSTs. LGB, HMD and RW helped in the interpretation of data and helped revise the manuscript. All authors read and approved the final draft.

## Pre-publication history

The pre-publication history for this paper can be accessed here:


